# Global Perspectives on Perinatal HIV and Its Implications for Sexual and Reproductive Health: A Scoping Review

**DOI:** 10.1177/23259582261432457

**Published:** 2026-06-16

**Authors:** Bandile Ernest Ndlazi, Regina Mampekase Molete, David Ditaba Mphuthi, Memme Girly Makua

**Affiliations:** 1 Department of Health Studies, 56866 University of South Africa, Pretoria, South Africa

**Keywords:** adolescents, perinatal HIV, sexual and reproductive health, HIV stigma, sexuality education

## Abstract

Globally, the management of adolescents and young people living with HIV has predominantly emphasized clinical outcomes, neglecting their overall well-being and sexual and reproductive health (SRH), making it essential to address these gaps for effective targeted interventions. Existing literature elucidating the SRH experiences of adolescents and young adults (people) living with perinatally acquired HIV (AYA-PHIV) across various countries was synthesized by this scoping review. We conducted a systematic search utilizing electronic database. Fifteen peer-reviewed articles met the inclusion criteria. The review identified 5 key themes in SRH research for AYA-PHIV. These include sexuality and social norms, HIV status disclosure, HIV-related stigma and social isolation, gaps in knowledge and access to SRH education, as well as barriers to healthcare access. To improve the SRH and wellbeing of AYA-PHIV, it is essential to implement targeted interventions including promoting SRH discussions, peer support, and adolescent-friendly facilities.

## Plain Language Summary Title

The dynamics of HIV and sexual and reproductive health for children and young adults born with HIV

Plain Language Summary

Around the world, providing care to adolescents and young people living with HIV has predominantly emphasized the traditional HIV treatment response, with limited attention to their general well-being, particularly the sexual and reproductive health (SRH). Addressing these gaps is essential for developing targeted interventions and effectively designing adolescent programs that meet the comprehensive SRH requirements of this population. This scoping review examined and analyzed the research findings of the studies conducted on SRH needs of adolescents and young adults born with HIV. We conducted a systematic search of research studies, utilizing electronic databases such as PubMed, Scopus, Web of Science, and Google Scholar. The search was done between February and July 2025 by a group of researchers. After an extensive search, deduplication, and shortlisting, 14 peer-reviewed articles met the inclusion criteria. To streamline the search and identify original research, the team of researchers decided to exclude Review papers, unpublished work and that did not focus exclusively on adolescents and young adults born with HIV. The review identified 5 emergent and repeated themes that characterize the research on SRH for adolescent and young adults born with HIV. These themes include sexuality and social norms, HIV status disclosure, HIV-related stigma and isolation, gaps in knowledge and access to SRH education, as well as barriers to healthcare access. Shared challenges faced by adolescents and young adults born with HIV were identified, including HIV stigma, family dynamics, healthcare access, peer pressure, and educational challenges. The researchers, based on these findings, concluded that targeted interventions are essential to enhance SRH for this group.

## Introduction

Children, adolescents, and young adults who acquired HIV vertically encounter unique challenges that impact different aspects of their lives and health. Globally, the United Nations Children's Fund estimated that approximately 3.2 million adolescents aged 10 to 24 were living with HIV in 2024, accounting for 8% of all people living with HIV.^1^ This population encounters unique challenges that influence societal dynamics, public policy, and health service delivery, particularly concerning their sexual health, reproductive health, and mental well-being. A comprehensive understanding of the existing evidence on sexual and reproductive health (SRH) needs of this group is essential. Such knowledge may contribute to the formulation of tailored interventions designed to ensure that adolescents and young adults living with perinatally acquired HIV (AYA-PHIV) receive the necessary support and resources for SRH.^2^

Growing up with HIV necessitates ongoing support and sharing of information regarding various aspects of transition. For adolescents growing up with vertically acquired HIV, sexual education is crucial as they navigate their unique circumstances.^3^ During this developmental stage, they often grapple with questions about the circumstances that led to their infection and how to manage their reality. However, several barriers impede effective communication between themselves, healthcare professionals and parents to share insights around SRH and sexuality.^4^ Social, cultural, and religious norms and beliefs create significant obstacles, making it difficult to share and receive the correct and reliable information.^4,5^ The stigma associated with discussing sexual matters at home can lead to the misconception that a young person is either thinking or already sexually active.^6^ These circumstances result in the use of other sources for information, such as peers, social networks, and online platforms.^7^ Creating another layer of complication, as the accuracy and appropriateness of such remains questionable.

The AYA-PHIV have sexual needs and desires from childhood.^8^ They also want to feel and be treated normally. This includes opportunities for equal access to information and services pertaining to their SRH. Despite societal expectations that they refrain from sexual activities, they do engage in such activities and need comprehensive information to protect themselves and their partners.^9,10^ They often aspire to be in romantic relationships and consider having children.^11^ This adds to the challenge of disclosing their status to potential partners. To navigate issues related to SRH, disclosure, stigma, and overall health and well-being, adolescents and young people living with HIV (AYPLHIV) in general should have access to information.^7,12,13^ This highlights the urgent need to improve their knowledge and self-care regarding sexuality, contraception, pregnancy, and prevention of sexually transmitted infections.^5,8–14^

The intersection of HIV and SRH is complex and multifaceted. The AYPLHIV often encounter stigma and discrimination which significantly hinders their access to health services and information.^15^ The stigma often emanates from family, healthcare settings, and social spaces.^16^ In some instances, this leads to many AYPLHIV avoiding healthcare facilities for fear of their HIV status being exposed.^17,18^ As they begin to explore intimate and sexual relationships, disclosure challenges become apparent with many fearing gossip and rejection.^11,19^ Additionally, negotiating safe sex practices and accessing contraceptive methods may be a challenge. These barriers can result in adverse SRH outcomes, further complicating their already precarious health situation.

Despite the noted challenges, there is a significant gap in understanding the SRH needs and experiences globally. Healthcare providers must be equipped to support AYPLHIV, including AYA-PHIV, yet cultural clashes often hinder effective communication.^20^ Access to SRH education and services varies significantly between low-middle-income countries and high-income countries.^21,22^ While some countries report adequate training for healthcare providers, other countries highlight training gaps and negative attitudes as barriers to care.^23^ Various approaches have inclusive ways of addressing cultural contexts within which behavior occurs. Addressing these challenges requires removing policy barriers and understanding the belief system, while actively involving all community members in the development of responsive SRH programs.^5,18–24^

This scoping review is warranted to synthesize existing literature on AYA-PHIV and SRH needs and experiences, to identify research gaps, and to inform future interventions aimed at improving SRH outcomes for this population. By examining the available literature on SRH needs of this group across diverse contexts, this review aims to provide valuable insights into the SRH challenges and limitations faced by this population group, as well as how healthcare systems can better support SRH needs.

## Methodology

The methods for this scoping review were guided by a previously published protocol.^25^

### Search and Identification of Literature

A systematic search was conducted across multiple databases, including PubMed, Scopus, Web of Science, and Google Scholar, between February 2025 and July 2025. To streamline our search and ensure relevance, we employed a set of targeted keywords such as “young people living/ born with HIV,” “sexual reproductive health,” “vertical HIV/ transmission,” “maternally acquired,” “perinatal HIV,” “born with HIV,” “stigma,” and “healthcare access,” “family planning,” “contraceptives,” “sex education,” “sexuality,” and “growing up with HIV.” We included studies published between January 2001 and June 2025 that specifically addressed SRH issues among AYA-PHIV.

### Eligibility Criteria

The search and selection process was structured around the population, concept and context (PCC) framework.^26^ The selection criteria encompassed articles published in peer-reviewed journals, from any country, written in English and classified as original research articles. The primary focus was published studies on adolescents and young adults living with HIV with age ranges of 10 to 30, with an emphasis on findings related to SRH for AYA-PHIV. The age 10 to 30 inclusion criteria was determined considering the global SRH focus and target of individuals up to 30 years of age. The selection studies considered various study designs, incorporating both quantitative and qualitative studies, with basic qualitative content analysis applied to analyze the findings.

### Study Selection

The scoping review was conducted and reported following the Preferred Reporting Items for Systematic Reviews and Meta-Analysis extension for Scoping Reviews (PRISMA-ScR) guidelines.^26,27^ A comprehensive literature review was performed by 2 independent reviewers (BN & RM) across the specified databases resulting in an initial identification of 269 studies. From this, a manual prescreening process was undertaken, resulting in the identification of 180 articles that met the preliminary selection criteria. The articles were subsequently uploaded to a shared reference management software, EndNote 20.^28^ Full-text screening was facilitated using Rayyan, a software application designed for systematic and scoping reviews.^29^ The 180 articles were imported into Rayyan for deduplication, followed by both abstract and full-text screening. After the removal of duplicates, 160 articles remained for further review. On completion of full-text review, 27 articles were deemed eligible. Of these, 10 articles were selected by all reviewers, while 17 were selected by only one reviewer. Discrepancies regarding the 17 studies were addressed through discussion among the 4 reviewers, with articles included only upon mutual agreement; ultimately, a consensus was reached on 5 articles. In total, 15 articles were identified and captured in the accompanying table of this article. Studies were excluded based on the following reasons:

- Unpublished studies to enhance the credibility, transparency, and reproducibility of the review conclusions
- Lack of specification regarding whether the study population was living with perinatally or vertically acquired HIV
- Focus on SRH for AYA-PHIV while the study population consisted of parents or healthcare providers.
- Age of study participants not aligned with the review's inclusion criteria
- Emphasis on sexuality and sexual behavior rather than sexual health.

### Data Extraction and Analysis

A deductive data extraction approach was applied, following the PCC framework and the agreed-upon terms of reference, utilizing a standardized extraction tool for data capture.^30^ Descriptive and exploratory data were extracted, encompassing bibliographic details of the studies, characteristics, and concepts related to SRH, the contexts in which these concepts are identified, key findings and gaps.^30^ The reviewers collaboratively created a codebook, which was subsequently used to conduct qualitative content analysis to identify common themes and subthemes across studies. This initial analysis was followed by a team discussion to reach a consensus on the final themes, enhancing the reliability and validity of the thematic analysis. A 6-step coding reliability thematic approach guided further analysis, beginning with code generation based on study objectives and preliminary themes. The qualitative data were analyzed by developing codes from the list generated from the study objectives and identified themes. Subsequently, the codes were categorized to facilitate effective clustering of text segments, highlighting emergent themes.

## Results

### Characteristics of the Included Studies

The studies that were identified and deemed eligible during the selection process are listed in detail in Table 1. Among the 15 studies identified, 3 were conducted in East African countries: Kenya, Malawi, and Tanzania. Additionally, 3 studies originated from North America, in the United States. In East Asia, 2 studies were conducted in China and Thailand, while another study from South Asia was conducted in India. In Southern Africa, 3 studies were carried out in South Africa. In West Africa, 2 studies were conducted in Côte d’Ivoire and Senegal. Lastly, one study was conducted in Western Europe, in France.

**Table 1.** Summary of Aims, Methods, Participants, and Key Findings of Studies Included in the Review.

Authors	Year of publication	Country	Aim/purpose	Study design	Study population	Key findings
Ndlazi et al^31^	2025	South Africa	To explore and describe the SRH barriers for young people living with vertically acquired HIV	Qualitative design	16 adolescents with vertically acquired HIV, receiving ART, age range 18-24	Discomfort with dating, limited family support, and negative sexual experiences. Participants were hesitant to disclose their HIV status due to fear of rejection. Family support was primarily emotional, lacking focus on SRH needs. Healthcare providers often overlook individual sexual health needs, resulting in limited client-focused services.
Bergam et al^32^	2022	South Africa	To evaluate how the mHealth intervention influences sexual health knowledge and behaviors in adolescents with perinatal HIV	Randomized Control Trial	80 adolescents with perinatally acquired HIV, receiving ART and fully aware of their HIV status, age range 15-19	Discomfort with discussing SRH at home due to family norms that discourage open dialogs about sexuality. Inadequate and stigmatizing SRH education from parents and caregivers. Adolescents first learn about SRH as teenagers in school through nontargeted and negative ways. Had to seek more clarity from peers and the internet than from clinicians and caregivers. Adolescents turned to technology, including pornography and social media for information about sex.
Gitahi et al^33^	2020	Kenya	To address the existing knowledge gaps in the postdisclosure experiences and psychosocial needs of older adolescents living with HIV	Qualitative Design: Focused group discussions (FDGs)	48 perinatally infected adolescents assigned to 8 FDGs, age range 16-19	Timing and circumstances for disclosure were often unplanned and triggered by health situations and in some instances accidental self-discovery through medical records. Concerns about how the status will affect future relationships and disclosure. Anticipated stigma was found to be a major barrier toward adherence and coping with HIV status. Desire for direct communication about HIV. In most cases, caregivers or siblings with a similar status were a source of social support.
Laborde-Balen et al^34^	2023	Senegal	Exploring how adolescent girls born with HIV who live outside of Dakar experience sexuality, what socio-health constraints they face and what support they receive from the healthcare system.	Anthropological design	40 HIV-positive children and adolescent girls. Age range 12-19	Girls from rural settings are confronted with the silence that surrounds sexuality and HIV. Stigma arising from premarital activity and the implications for family horror. Fear of disclosing HIV status to avoid stigma and shame affects social interaction and healthcare engagement. Ambivalence of healthcare workers regarding providing SRH services to adolescents due to moral beliefs. Limited discussions around SRH among healthcare professionals. Influence of cultural beliefs on health behaviors.
Lolekha et al^35^	2015	Thailand	To assess knowledge, attitudes and practices of perinatally HIV infected youth and youth reporting sexual risk behaviors	Survey	197 youth living with HIV, age range 11-18	Half of the participants showed a lack of family planning, reproductive health and STI knowledge. Schools, healthcare providers, and caregivers were identified as primary sources of sexual health education. Girls had more positive attitudes toward safe sex and risky behavior than boys. 5% reported to have engaged in sexual intercourse with about a third reporting risk behaviors. Low condom usage and family planning practices increase the risk of HIV and STI transmission to sexual partners.
Mu et al^36^	2015	China	To assess SRH and HIV knowledge and perceptions among perinatally infected adolescents	Descriptive cross-sectional design	124 adolescents living with perinatally acquired HIV, age range 10-19	Almost 80% of participants had never discussed puberty development and sexuality with their parents. Over 50% of participants had never heard of condoms, and 20% reported not having SRH information sources or knowledge. Adolescents older than 15 who had been disclosed of HIV status were more likely to seek and acquire more knowledge.
Mwalabu et al^37^	2017	Malawi	Explore the sex and relationship experiences of young women growing up with perinatally acquired HIV to understand how to improve SRH care and associated outcomes	Qualitative design: Case study	14 cases of young women living with perinatally acquired HIV. Age range 15-19	Desire for normalcy despite the HIV status. Young women reported becoming sexually active for multiple reasons, as some sought a sense of love, intimacy, acceptance and belonging as they lacked this at home or within their peer groups. Cultural silence surrounding sexual health with religious norms emphasizing abstinence, created ambivalence among caregivers and healthcare providers to discuss sexual health openly. Power dynamics in relationships affecting as for others; their sexual activity was more functional, to meet survival needs while others expressed little or no control over negotiating safer sex or contraception. Caregivers and nurses viewed the sexual activity from the clinical perspective, fearing onward transmission of HIV and advocating abstinence or condoms where possible.
Tisseron et al^38^	2024	Cote d’Ivoire	To explore the sexual knowledge, practices and needs for adolescents living with HIV.	Qualitative design	Adolescents living with HIV acquired perinatally 18 (9 male and 9 female) without previous pregnancies and an additional 8 who became pregnant. Age range 15-19	Fear and anxiety about sexual activity, including concerns of possibly transmitting HIV to partners. The taboo nature of sexuality resulted in difficulty in talking about SRH with parents and healthcare professionals, while turning to friends for advice. Desire for improved SRH communication by healthcare providers. One-third of female participants experienced nonconsensual sex and sexual violence. Low level of condom use despite knowledge. Reasons for low condom use include difficulty in negotiating for girls and an undetectable viral load.
Vranda et al^39^	2018	India	To examine the SRH needs and concerns of adolescents living with perinatally infected HIV in India.	Qualitative design	20 adolescents living with perinatally acquired HIV and aware of their status	Desire for romantic relationships as adolescents reported challenges with romantic relationships and were unsure whether to marry a person living with HIV or HIV-negative. They aspire to have children but are worried about transmitting the virus to their unborn children. Discomfort with discussing sex with parents. Had concerns with delayed puberty and found it difficult to discuss these with their mothers. Sexuality information was sought from friends. Internalized stigma and insecurity impacted adolescents’ self-esteem and confidence.
Comfort et al^40^	2022	New York	To describe the sexual and reproductive perceptions, goals and needs of adolescents living with HIV	Qualitative design	25 female adolescents living with HIV, 14 were perinatally acquired. Age range 17-25	Lifelong relationship with healthcare providers. Fears regarding disclosure and experience, along with expectations of rejection, judgment and persecution. The value of accessing antiretroviral therapy to maintain general, sexual, and reproductive health
Ibrahim et al^41^	2024	France	To explore the sexual health of young adults living with perinatally acquired HIV	Qualitative design	25 young adults living with perinatally acquired HIV were diagnosed before reaching age 13. Are range 18-25	HIV and sexuality subjects were described as dual taboo topics in most families. Participants had to self-educate through information gathered from school and websites. Fear of transmitting HIV to sexual partners
Fair et al^42^	2012	USA	To explore challenges confronted by AYA living with perinatal HIV in forming and maintaining intimate, romantic relationships	Quantitative design	35 adolescents and young adults living with perinatally acquired HIV, who were either current or former clients at the 2 study sites in the Southeastern United States. Age range 15-30	Participants who disclosed their HIV status to partners described their HIV as often referred to as a secret and mostly associated with rejection. Disclosure was also linked to the participant's experience with own diagnosis. Intentionally delaying dating due to HIV.
Woollett et al^43^	2021	South Africa	To explore adolescents living with HIV experiences and recommendations for a responsive healthcare system in adolescence	Qualitative design	25 adolescents living with HIV on HIV treatment and know their HIV status (disclosed to) and are retained in care	Adolescents felt they would be more comfortable in facilities that cater only for them and highlighted the need for youth friendly facilities and attitudes. Discrimination due to a limited understanding of vertical transmission. Inadvertent disclosure and discrimination in the adult clinics.
Busza et al^44^	2013	Tanzania	To explore the perceptions and experiences of vertically infected adolescents on sex, relationship, and health.	Qualitative design	14 adolescents, 12 family members and 12 home-based care providers. Adolescents age range 15-19	Adolescents claimed they were too young to think about sex and were also worried about having to disclose their status to prospective partners, with the fear of rejection. The desire to have a stable intimate relationship and children was found among adolescents.
Fair et al^45^	2016	USA	To describe self-reported SRH information and services received by adolescents and young adults with perinatally acquired HIV in clinical settings and identify areas of unmet needs	Quantitative design	35 adolescents and young adults living with perinatally acquired HIV, who were either current or former clients at the 2 study sites in the Southeastern United States. Age range 15-30	Sexual health discussions were found in healthcare settings, with sexual risk reduction and pregnancy prevention the most addressed topics. Males reported fewer SRH discussions than females. Participants were mostly comfortable discussing SRH with healthcare providers.

The review identified 5 themes that characterized SRH needs among AYA-PHIV: sexuality and social norms, HIV status disclosure, stigma and isolation, knowledge gap, and SRH education access and barriers to healthcare. Although the studies were conducted across various continents and diverse countries, the identified themes were remarkably consistent.

### Sexuality and Social Norms

Five studies examined sexuality and social norms influencing adolescent behavior, particularly among those born with HIV. Findings indicate that societal expectations and gender norms significantly influence attitudes toward adolescents’ sexuality, also revealing differences in how parents view these issues.^34,38^ Adolescent girls, in particular, experience pressure to conform to norms that emphasize virginity and abstinence. This pressure creates uncertainty among caregivers and healthcare providers about how to approach discussions on sexual health.^34,37^

Cultural beliefs play a significant role in shaping adolescents’ perception of their sexual health needs, relationships, and societal expectations regarding sexual behavior.^34,37^ The stigmatization of premarital sexual activity adversely affects self-esteem, relationships, and access to SRH resources and services. Adolescent girls often fear being perceived and labeled as promiscuous, which leads to secrecy and internal conflicts.^36,37^ Additionally, cultural silence and the taboo surrounding sexuality hinder open discussions, resulting in feelings of isolation and reinforcement of misinformation and fear.^37,38^ Resistance to external influences promoting nontraditional views on sexuality is also prevalent, limiting the effectiveness of interventions.^32,34^

### HIV Status Disclosure

Three studies indicate that AYA-PHIV often learn about their HIV status through unplanned and distressing circumstances, frequently due to medical complications. The caregivers are usually forced to disclose to adolescents under pressure due to advanced illness, which adversely affects their mental health.^33,34,39^ Families often maintain secrecy regarding the child's HIV status while employing strategies such as hiding medication and or fabricating narratives to avoid stigma and shame.^34^ Self-discovery of one's status is particularly common among adolescent males, who may encounter this information through medical records or educational materials.^33^ Later in life, postdisclosure experiences vary as some feel empowered to manage their health while others struggle with feelings of betrayal and anger toward their parents.^33^ Support systems comprising peer and family members are vital in alleviating emotional distress related to disclosure.

Nine studies identified challenges with status disclosure to sexual and intimate partners.^12–15,31^ Despite the challenges associated with disclosure, AYA-PHIV understanding of their HIV status is the first layer of disclosure, followed by disclosure to sexual and intimate partners. The findings also suggest that HIV stigma experience and partner maturity influence adolescents’ decisions about disclosure, particularly in intimate relationships where fear of rejection is prevalent.^39–42^ Notably, the prevalence of nondisclosure to partners raises concerns regarding mental health and relationship dynamics.^35,36^ In most instances, adolescents avoided and delayed engaging in intimate relationships due to fear of disclosure with its presumed complications.^31,40–42,44^

### Stigma and Isolation

Collectively, findings from 8 studies indicate that societal stigma contributes to feelings of isolation among AYA-PHIV. This isolation negatively affects their mental health and diminishes their willingness to seek healthcare and socially engage.^34,35,37–39^ Adolescents worry about how their HIV status may affect future relationships and societal acceptance.^31,32^

The external stigma often results in internalized stigma, leading to low self-esteem, reluctance to disclose as well and anxiety toward relationships.^33^ This often results in self-isolation from social support networks due to fears of judgment from peers and potential rejection and abandonment.^34,35,40–42^

### Knowledge Gaps and SRH Education Access

Across studies, significant gaps in SRH education were identified that adversely affect adolescents born with HIV's ability to make informed decisions.^34,35,37–39^ Parental discomfort, cultural taboos, and privacy issues limit discussions about sexuality and HIV within families.^41^ As a result, AYA-PHIV miss opportunities to ask relevant questions about SRH, as negative and threatening messaging is used to instill fear rather than promoting healthy lifestyles.^32,36^ Consequently, many of these adolescents rely on technology (social media, pornography, and other internet sites) and peer support from friends as an alternative source of SRH knowledge, which often leads to misinformation and peer pressure.^32,36,38,39,41^ Contrarily, the Thailand study identified healthcare providers and primary caregivers as sources of sexual education,^35^ highlighting the need for comprehensive sexual education in these settings.

Other studies show that peer support groups and associations for people living with HIV provide important emotional support.^33,43^ They offer education and counseling, fostering health discussions about relationships and sexuality, which helps adolescents to effectively navigate their sexual needs.^32,33,34,36–39^ Digital interventions such as mobile health programs have been reported to be effective in improving SRH knowledge and self-confidence among adolescents. One of the studies found that these programs empower adolescents born and living with HIV, though some still seek clarity on certain SRH topics.^32^

### Barriers to Access to Healthcare

Five studies reveal significant disparities in access to comprehensive SRH services, including contraception and prevention services, particularly among adolescent girls in urban and rural settings.^34,37,38,43,45^ Adolescents living with HIV encounter barriers such as distance to healthcare, stigma, and healthcare provider attitudes, which often prioritize HIV management over broader SRH concerns.^31,34,35,40–45^ This leads to inadequate and stigmatizing education and forces AYA-PHIV to navigate multiple facilities to avoid judgment, disrupting care continuity and negative health outcomes.^34,37^ They prefer youth friendly services that integrate HIV, SRH and mental health support in a one stop shop model.^4–45^ Another dimension that hinders effective communication is the emotional connection with healthcare providers. The AYA-PHIV often view these providers as parental figures, which can hinder open discussion about sexual matters.^38^

## Discussion

The scoping review synthesized existing literature elucidating the SRH experiences of AYA-PHIV across various countries. Despite the presence of numerous studies conducted on AYPLHIV, the review identified only 15 studies from 12 distinct countries that specifically focused on AYA-PHIV. Through this analysis, 5 themes emerged: sexuality and social norms, HIV status disclosure, stigma and social isolation, knowledge gaps, and SRH education and barriers to access to healthcare. The findings from the review underscore critical gaps that must be addressed in program planning, policy influence, and future research.

Adolescents and young adults living with perinatally acquired HIV must navigate a complex of societal expectations, personal desires and stigma. Across the identified studies, a recurring theme is the tension between cultural norms and individual sexual agency, needs, and aspirations, which leads to significant societal pressures surrounding sexuality, particularly concerning issues of virginity and abstinence.^34,37^ As a result, these adolescents, especially AYA-PHIV, frequently encounter challenges in initiating discussions about their SRH concerns due to the perception that such topics are unimportant for this group. The persistent association of HIV with promiscuity exacerbates stigma, ultimately discouraging adolescents from seeking assistance, leading to potentially severe health complications.^46^ This situation often culminates in gender inequality and discrimination that restricts their autonomy regarding SRH-related decisions.^5,47^ Cultural influences impose stricter regulations on adolescent girls, further limitating their autonomy. This is evident from findings that indicate the prevalence of stigma associated with premarital sexual encounters, which ultimately has detrimental effects on individuals’ self-esteem and interpersonal relationships.^34^ For AYA-PHIV, the dual stigma of HIV and SRH complicates their situation. Cultural beliefs not only influence behavior but also shape adolescents’ understanding of their sexual health needs.^37^ Understanding diverse cultural perspectives is crucial, highlighting the need for culturally sensitive education and support tailored to AYA-PHIV. Assigning gender-specific healthcare providers may also help facilitate discussions for those uncomfortable with mixed gender interaction.

Disclosure remains a pivotal moment in the lives of AYA-PHIV, shaping their self-perception, relationships, and their access to care. This review highlights the challenges of HIV status disclosure and its subsequent implications, often complicated by fears of stigma and shame within families.^34^ Adolescents often learn about their HIV status in unanticipated and distressing ways, leading to feelings of betrayal when they discover it independently due to family secrecy.^34,48,49^ While some parents encourage status disclosure, the predominant tendency is to encourage secrecy to protect the child and their siblings.^45^ Secrecy becomes part of their lives as they also continue keeping their HIV status private.^50^

As adolescents start engaging in intimate relationships, concerns surrounding disclosing their HIV status become increasingly pronounced.^9,31,35^ Disclosure is critical for self-perception and relationships; however, fear of rejection and public persecution often inhibits them from sharing this information with partners.^40,51,52^ Additionally, delayed disclosure by parents or caregivers can adversely affect treatment adherence, overall mental well-being, as well as social interactions, while postponing discussions about SRH.^48,53^ This emphasizes the importance of age-appropriate disclosure process to better prepare adolescents.

Effective disclosure depends on empowerment and adequate information. Studies indicate that adolescents possessing greater HIV knowledge are more likely to disclose their status and understand its benefits while being able to assess appropriate people for such disclosures.^13,54^ Support from peers and family members is vital in assisting adolescents in navigating the emotional complexities of disclosure. This underscores the importance of developing and participating in peer support groups and other support structures available in their respective countries to aid in the disclosure process.^13^

HIV stigma significantly impacts the emotional and social experiences of AYA-PLHIV. It fosters feelings of isolation, adversely impacting their mental health, and willingness to seek care.^15,46^ Concerns regarding future relationships exacerbate internalized stigma, resulting in low self-esteem and hesitance to disclose their HIV status.^34^ The absence of open dialog about HIV within families intensifies this isolation.^33^ This highlights the necessity for candid conversations and practical guidance that is integrated into the routine care as adolescents navigate different life stages.

A study in South Africa found there was no common understanding of the different ways people acquire HIV, particularly perinatal acquisition, which contributes to the perpetuation of stigma and hinders disclosure efforts.^43^ Limited knowledge about HIV, past experiences of rejection, lack of HIV testing and prevalent myths about HIV contribute to stigma.^55^ If not managed, HIV-related stigma can detrimentally affect the quality of life of adolescents living with HIV, regardless of the mode of acquisition.

Substantial gaps still exist in SRH knowledge among AYA-PHIV, which impedes informed health decisions. Enhancing social support, fostering social influence and collaboration among individuals, families, and healthcare providers can improve adolescents’ engagement with healthcare services.^7^

This review highlights that healthcare providers predominantly concentrate on HIV management while overlooking broader SRH concerns. When SRH is addressed, the education provided is often inadequate, abstinence based, and stigmatizing, reflecting a limited capacity for comprehensive care.^31,32,33,[Bibr bibr34-23259582261432457]–35,39–51^ Contrary to this finding, other studies report that adolescents living with HIV are more likely to seek sexual health information from healthcare providers rather than older family members.^48,56^ This underscores the importance of healthcare provider characteristics and the development of trusting relationships over time. Beyond HIV management, AYA-PHIV need essential skills such as self-monitoring, planning, goal setting, and health evaluation to foster self-management and overall well-being.^7^

Parental discomfort in discussing sexuality limits meaningful engagements, leading many adolescents to resort to unreliable sources such as social media and other internet based sources for SRH, resulting in misinformation.^16^ Parents often hesitate to talk about sexual and intimate relationships with young people, regardless of their HIV status. The reluctance, coupled with feelings of judgment and control, drives adolescents to seek information privately. Adolescents prefer obtaining information in nonjudgmental environments, making them more likely to search on the Internet than approach an adult.^7,57^ Other studies corroborate the limited communication between caregivers and adolescents regarding SRH.^51,58^

This review highlights schools as critical avenues for disseminating SRH information,^35^ paralleling findings from a study conducted in a country named Lao People's Democratic Republic.^47^ Adolescents felt more at ease discussing sexuality with Life Orientation educators in schools that provided comprehensive information beyond abstinence-only messaging.^56^ These insights emphasize the necessity for integrating comprehensive sexual education within both school curricula and healthcare settings.

While informal peer support networks can offer emotional support, they also pose risks of the dissemination of misinformation, highlighting the need for structured educational programs that provide accurate knowledge. The AYA-PLHIV require systems that acknowledge their psychosocial and mental health needs to enhance resilience while improving their SRH understanding.^56^ The review suggests that formalized support groups, associations for people living with HIV and peer educators can play pivotal roles in providing emotional support and facilitating healthy discussions about relationships and sexuality.^34,38^ Participation in peer support groups has been associated with increased awareness of available SRH services.^53^ To effectively address these needs, youth friendly service centers should be established to provide comprehensive sexuality education, reproductive health services and counseling in a confidential and accessible environment.^33,51^

The review indicates that disparities in access to comprehensive SRH persist for adolescents born and growing up with HIV, particularly among girls in both urban and rural settings. Adolescents from rural settings face limited access to healthcare services and information, resulting in inadequate knowledge of essential SRH aspects.^5^ Key barriers include geographical distance from healthcare facilities and stigma associated with HIV and SRH issues.

Studies have demonstrated that the provision of SRH information and support by healthcare providers is insufficient, leaving adolescents uncertain about what to expect regarding engagement beyond HIV management.^7,52^ This uncertainty may contribute to underutilization of healthcare providers for SRH guidance, suggesting that provider characteristics can act as a barrier.^51–59^

Many healthcare providers lack the necessary skills and training to effectively address the unique SRH needs of AYPLHIV, while some of the trained simply lost interest due to workload.^51,59^ There is an urgent need for training programs that address provider attitudes toward young people's sexuality as well as the specific SRH needs and rights of AYPHIV, with more attention given to AYA-PHIV.^51,59^ The AYA-PHIV express a strong preference for integrated youth friendly services that encompass HIV, SRH, and mental health support to improve their SRH and well-being.

### Review Limitations

Despite the efforts to conduct a comprehensive search of published studies, our search was limited to the 4 most utilized data bases in public health research. Consequently, relevant unpublished studies and articles in languages outside the inclusion criteria may have been missed, potentially introducing selection bias. Despite the listed limitations, the findings presented in this review add some invaluable insights on the SRH challenges faced by AYA-PHIV.

## Conclusion

The thematic analysis underscores the shared experiences of AYA-PHIV across diverse cultural contexts, particularly challenges related to stigma, family dynamics, healthcare issues, peer influence, and educational gaps while also acknowledging unique cultural factors that may influence these experiences. The findings highlight the importance of age appropriate disclosure for children and adolescents living with HIV. To enhance the well-being of AYA-PHIV, targeted interventions are essential, including fostering open dialogs about sexuality within families and communities. Adolescents’ friendly clinics that provide integrated SRH and HIV services remains a critical gap in many settings while ensuring privacy and nonjudgmental interactions with healthcare providers and patients continue to offer emotional support and accurate health information.

## Supplemental Material

sj-rtf-1-jia-10.1177_23259582261432457 - Supplemental material for Global Perspectives on Perinatal HIV and Its Implications for Sexual and Reproductive Health: A Scoping Review Supplemental material, sj-rtf-1-jia-10.1177_23259582261432457 for Global Perspectives on Perinatal HIV and Its Implications for Sexual and Reproductive Health: A Scoping Review by Bandile Ernest Ndlazi, Regina Mampekase Molete, David Ditaba Mphuthi and Memme Girly Makua in Journal of the International Association of Providers of AIDS Care (JIAPAC)
