# Supplementary material for: Global Perspectives on Perinatal HIV and Its Implications for Sexual and Reproductive Health: A Scoping Review
Source: J Int Assoc Provid AIDS Care. 2026 Jun 16;25:23259582261432457. doi: 10.1177/23259582261432457 (PMC13272983; doi:10.1177/23259582261432457)
Supplement: sj-rtf-1-jia-10.1177_23259582261432457 - Supplemental material for Global Perspectives on Perinatal HIV and Its Implications for Sexual and Reproductive Health: A Scoping Review [file sj-rtf-1-jia-10.1177_23259582261432457.rtf]

Supplementary Figures/ Tables 

1.	Fig. 1 Flow diagram of study selection


Adapted from: Page MJ, McKenzie JE, Bossuyt PM, et al. The PRISMA 2020 statement: an updated guideline for reporting systematic reviews. BMJ. 2021; 372:n71. DOI: 10.1136/bmj.n71.


2.	Table 2: Preferred Reporting Items for Systematic reviews and Meta-Analyses extension for Scoping Reviews (PRISMA-ScR) Checklist25
SECTION	ITEM	PRISMA-ScR CHECKLIST ITEM	REPORTED ON PAGE #	
TITLE	
Title	1	Identify the report as a scoping review.	Page 1	
ABSTRACT	
Structured summary	2	Provide a structured summary that includes (as applicable): background, objectives, eligibility criteria, sources of evidence, charting methods, results, and conclusions that relate to the review questions and objectives.	Page 1	
INTRODUCTION	
Rationale	3	Describe the rationale for the review in the context of what is already known. Explain why the review questions/objectives lend themselves to a scoping review approach.	Page 3	
Objectives	4	Provide an explicit statement of the questions and objectives being addressed with reference to their key elements (e.g., population or participants, concepts, and context) or other relevant key elements used to conceptualize the review questions and/or objectives.	Page 4	
METHODS	
Protocol and registration	5	Indicate whether a review protocol exists; state if and where it can be accessed (e.g., a Web address); and if available, provide registration information, including the registration number.	Not applicable	
Eligibility criteria	6	Specify characteristics of the sources of evidence used as eligibility criteria (e.g., years considered, language, and publication status), and provide a rationale.	Page 4	
Information sources*	7	Describe all information sources in the search (e.g., databases with dates of coverage and contact with authors to identify additional sources), as well as the date the most recent search was executed.	Page 4	
Search	8	Present the full electronic search strategy for at least 1 database, including any limits used, such that it could be repeated.	Page 3	
Selection of sources of evidence†	9	State the process for selecting sources of evidence (i.e., screening and eligibility) included in the scoping review.	Page 3-4	
Data charting process‡	10	Describe the methods of charting data from the included sources of evidence (e.g., calibrated forms or forms that have been tested by the team before their use, and whether data charting was done independently or in duplicate) and any processes for obtaining and confirming data from investigators.	Page 5	
Data items	11	List and define all variables for which data were sought and any assumptions and simplifications made.	Page 3	
Critical appraisal of individual sources of evidence§	12	If done, provide a rationale for conducting a critical appraisal of included sources of evidence; describe the methods used and how this information was used in any data synthesis (if appropriate).	Page 3	
Synthesis of results	13	Describe the methods of handling and summarizing the data that were charted.	Page 5	
RESULTS	
Selection of sources of evidence	14	Give numbers of sources of evidence screened, assessed for eligibility, and included in the review, with reasons for exclusions at each stage, ideally using a flow diagram.	Page 4-5	
Characteristics of sources of evidence	15	For each source of evidence, present characteristics for which data were charted and provide the citations.	Page 7	
Critical appraisal within sources of evidence	16	If done, present data on critical appraisal of included sources of evidence (see item 12).	Not applicable	
Results of individual sources of evidence	17	For each included source of evidence, present the relevant data that were charted that relate to the review questions and objectives.	Page 7-15	
Synthesis of results	18	Summarize and/or present the charting results as they relate to the review questions and objectives.	Page 7-15	
DISCUSSION	
Summary of evidence	19	Summarize the main results (including an overview of concepts, themes, and types of evidence available), link to the review questions and objectives, and consider the relevance to key groups.	Page 16-19	
Limitations	20	Discuss the limitations of the scoping review process.	Page 23	
Conclusions	21	Provide a general interpretation of the results with respect to the review questions and objectives, as well as potential implications and/or next steps.	Page 24	
FUNDING	
Funding	22	Describe sources of funding for the included sources of evidence, as well as sources of funding for the scoping review. Describe the role of the funders of the scoping review.	Not applicable 	
From: Tricco AC, Lillie E, Zarin W, et al. PRISMA Extension for Scoping Reviews (PRISMA-ScR): Checklist and Explanation. Ann Intern Med. 2018; 169(7): 467-73. DOI: 10.7326/m18-0850 
